# Supplementary material for: Toward an Affordable Density-Based Measure for the Quality of a Coupled Cluster Calculation
Source: J Phys Chem A. 2026 May 22;130(22):4289–98. doi: 10.1021/acs.jpca.6c00684 (PMC13244464; doi:10.1021/acs.jpca.6c00684)
Supplement: Supplementary file 1 [file jp6c00684_si_001.zip › ESI files/ESI Cover Page.pdf]

SUPPORTING INFORMATION FOR:

Toward an affordable density-based measure for the quality of a coupled cluster calculation

Gregory H. Jones,[1, a)] Kaila E. Weflen,[2, b)] and Jan M. L. Martin*[2, c)]

[1)] *Quantum Theory Project, Department of Chemistry, University of Florida, Gainesville, FL 32611, USA*

[2)] *Department of Molecular Chemistry and Materials Science, Weizmann Institute of Science, 7610001 Reḥovot, Israel.*

(Dated: Revision of `jp-2026-00684e`, 22 April 2026)

Contents

- `getINDbar2.py` Python program to extract the relevant diagnostics from CFOUR outputs
- `ESI_deltaINDdiagnostic.xlsx` Excel workbook with various static correlation diagnostics for the W4-17 dataset and different basis sets.

[a)] Electronic mail: gh.jones@ufl.edu

[b)] Electronic mail: kaila.weflen@weizmann.ac.il

[c)] Corresponding author: gershom@weizmann.ac.il
